# Supplementary material for: A Set of Dysregulated Target Genes to Reduce Neuroinflammation at Molecular Level
Source: Int J Mol Sci. 2022 Jun 28;23(13):7175. doi: 10.3390/ijms23137175 (PMC9266409; doi:10.3390/ijms23137175)
Supplement: Supplementary file 1 [file ijms-23-07175-s001.zip › ijms-1724386-supplementary.pdf]

Table S1. Top twenty up- and downregulated differentially expressed genes in HT22 and BV2 cells.

**HT22 top 20 upregulated genes**				
**Gene ID**	**Gene name**	**Base mean**	**log_2 fd**	**padj**
ENSMUSG00000029417	*Cxcl9*	1668.46	12.73	3.94e-52
ENSMUSG00000082292	*Gm12250*	242.46	9.94	1.33e-18
ENSMUSG00000054072	*Iigp1*	6173.24	9.13	4.96e-179
ENSMUSG00000073555	*Gm4951*	661.99	9.12	7.49e-54
ENSMUSG00000090942	*F830016B08Rik*	143.11	8.99	3.89e-21
ENSMUSG00000105504	*Gbp5*	355.34	8.91	1.78e-43
ENSMUSG00000092021	*Gbp11*	50.78	8.53	6.24e-15
ENSMUSG00000066363	*Serpina3f*	119.15	8.44	1.18e-17
ENSMUSG00000028037	*Ifi44*	2024.01	8.37	5.05e-150
ENSMUSG00000060183	*Cxcl11*	170.70	8.21	1.94e-42
ENSMUSG00000078853	*Igtp*	5277.10	7.94	2.71e-162
ENSMUSG00000078920	*Ifi47*	1254.50	7.90	1.05e-239
ENSMUSG00000063286	*Gm8995*	730.36	7.80	7.52e-20
ENSMUSG00000030107	*Usp18*	404.02	7.64	6.25e-40
ENSMUSG00000035186	*Ubd*	21.69	7.58	2.11e-11
ENSMUSG00000074896	*Ifit3*	262.35	7.57	1.02e-28
ENSMUSG00000035208	*Slfn8*	166.43	7.40	3.73e-23
ENSMUSG00000032690	*Oas2*	298.35	7.33	6.12e-24
ENSMUSG00000034438	*Gbp8*	224.03	7.30	1.31e-13
ENSMUSG00000073418	*C4b*	35.17	7.28	5.59e-15
**HT22 top 20 downregulated genes**				
**Gene ID**	**Gene name**	**Base mean**	**log_2 fd**	**padj**
ENSMUSG00000014813	*Stc1*	8.29	5.67	1.39e-04
ENSMUSG00000038578	*Susd1*	14.34	4.92	3.09e-06
ENSMUSG00000068874	*Selenbp1*	49.61	4.45	3.65e-06
ENSMUSG00000004791	*Pgf*	26.06	4.10	3.70e-03
ENSMUSG00000049871	*Nlrc3*	37.90	3.75	5.47e-03
ENSMUSG00000026271	*Gpr35*	18.40	3.73	6.94e-03
ENSMUSG00000074064	*Mlycd*	64.84	3.38	1.06e-02
ENSMUSG00000035711	*Dok3*	859.31	3.37	2.84e-04
ENSMUSG00000097203	*4732419C18Rik*	17.55	3.35	3.43e-02
ENSMUSG00000005824	*Tnfsf14*	22.83	3.34	1.04e-04
ENSMUSG00000040584	*Abcb1a*	30.13	3.17	4.91e-02
ENSMUSG00000022885	*St6gal1*	101.95	2.99	2.79e-03
ENSMUSG00000085165	*Gm12089*	11.60	2.97	7.56e-02
ENSMUSG00000040528	*Milr1*	699.63	2.97	1.43e-02
ENSMUSG00000031722	*Hp*	92.04	2.94	5.36e-14

ENSMUSG00000031785	*Adgrg1*	66.65	2.91	7.01e-03
ENSMUSG00000113035	*AC157822.3*	31.28	2.82	9.46e-02
ENSMUSG00000025383	*Il23a*	31.43	2.71	8.12e-02
ENSMUSG00000025347	*Mettl7b*	13.32	2.56	1.52e-02
ENSMUSG00000037411	*Serpine1*	368.60	2.54	6.22e-21
**BV2 top 20 upregulated genes**				
**Gene ID**	**Gene name**	**Base mean**	**log_2 fd**	**padj**
ENSMUSG00000075602	*Ly6a*	5582.61	11.31	1.50e-117
ENSMUSG00000054072	*Iigp1*	6173.24	10.66	7.34e-133
ENSMUSG00000034438	*Gbp8*	224.03	10.63	1.62e-27
ENSMUSG00000029417	*Cxcl9*	1668.46	10.40	4.71e-70
ENSMUSG00000073555	*Gm4951*	661.99	9.75	1.76e-68
ENSMUSG00000041481	*Serpina3g*	137.84	9.34	1.63e-22
ENSMUSG00000090942	*F830016B08Rik*	143.11	9.33	1.45e-21
ENSMUSG00000046031	*Fam26f*	84.48	9.31	3.06e-20
ENSMUSG00000066363	*Serpina3f*	119.15	9.20	5.17e-20
ENSMUSG00000079018	*Ly6c1*	1090.44	9.08	4.09e-43
ENSMUSG00000040264	*Gbp2b*	3661.68	9.07	7.64e-55
ENSMUSG00000028270	*Gbp2*	5034.36	8.39	1.97e-102
ENSMUSG00000060183	*Cxcl11*	170.70	8.39	1.84e-18
ENSMUSG00000039699	*Batf2*	361.43	8.25	2.87e-74
ENSMUSG00000079363	*Gbp4*	1341.60	8.24	6.65e-185
ENSMUSG00000022582	*Ly6g*	26.51	8.11	9.51e-14
ENSMUSG00000036594	*H2-Aa*	34.41	8.24	1.49e-13
ENSMUSG00000053318	*Slamf8*	181.21	7.99	2.80e-48
ENSMUSG00000105504	*Gbp5*	355.34	7.97	3.23e-65
ENSMUSG00000027514	*Zbp1*	343.78	7.93	1.52e-30
**BV2 top 20 downregulated genes**				
**Gene ID**	**Gene name**	**Base mean**	**log_2 fd**	**padj**
ENSMUSG00000026180	*Cxcr2*	201.98	7.01	3.68e-51
ENSMUSG00000030790	*Adm*	100.59	6.93	6.87e-19
ENSMUSG00000047786	*Lix1*	16.13	6.70	7.33e-08
ENSMUSG00000051212	*Gpr183*	12.53	6.58	8.36e-08
ENSMUSG00000024388	*Myo7b*	11.36	6.37	4.94e-07
ENSMUSG00000082361	*Btc*	8.73	5.59	2.36e-05
ENSMUSG00000003484	*Cyp4f18*	6.92	5.46	1.07e-04
ENSMUSG00000049130	*C5ar1*	69.65	5.42	4.54e-22
ENSMUSG00000040447	*Spns2*	6.78	5.40	1.06e-04
ENSMUSG00000028972	*Car6*	33.09	5.21	1.28e-06
ENSMUSG00000021792	*Fam213a*	11.49	5.00	5.17e-05
ENSMUSG00000026959	*Grin1*	7.24	4.93	3.48e-04
ENSMUSG00000028758	*Kif17*	10.06	4.84	9.60e-05

ENSMUSG00000027636	*Sla2*	5.14	4.76	1.19e-03
ENSMUSG00000038578	*Susd1*	14.34	4.73	1.30e-04
ENSMUSG00000029372	*Ppbp*	14.12	4.71	1.35e-04
ENSMUSG00000027221	*Chst1*	44.86	4.68	1.66e-05
ENSMUSG00000025329	*Padi1*	4.38	4.59	3.27e-03
ENSMUSG00000060470	*Adgrg3*	87.18	4.56	3.39e-24
ENSMUSG00000099098	*1110035H17Rik*	10.83	4.51	1.19e-04

Tables show gene ID, gene name, base mean (the mean of normalized counts of all samples, normalizing for sequencing depth), log_2 fd (fold change) and padj (adjusted p-value).

Table S2. Effect of inflammation and co-culture factors and their interaction.

**HT22 Upregulated**	**Co-culture**	**Inflammation**	**Interaction**
Cxcl9	F (1,8) = 27.00; p <0.0001	F (1,8) = 25.92; *p*<0.0001	F (1,8) = 27.00; *p*<0.0001
ligp1		F (1,8) = 24.92; *p*=0.0010	
Gbp5	F (1,8) = 25.92; p <0.0001	F (1,8) = 27.00; *p*<0.0001	F (1,8) = 24.92; *p*= 0.0010
Gbp11	F (1,8) = 16.51; *p*=0.003	F (1,8) = 24.92; *p*=0.0010	
Ifi44	F (1,8) = 25.92; *p*<0.0001	F (1,8) = 25.92; *p*<0.0001	F (1,8) = 27.00; *p*<0.0001
Cxcl11	F (1,8) = 27.00; *p*<0.0001	F (1,8) = 24.92; *p*=0.0010	F (1,8) = 27.00; *p*<0.0001
Igtp	F (1,8) = 27.00; *p*<0.0001	F (1,8) = 24.92; *p*=0.0010	F (1,8) = 27.00; *p*<0.0001
Ifi47	F (1,8) = 25.92; *p*<0.0001	F (1,8) = 25.92; *p*<0.0001	F (1,8) = 27.00; *p*<0.0001
Usp18		F (1,8) = 24.92; *p*=0.0010	F (1,8) = 17.00; *p*=0.0033
Ifit3		F (1,8) = 24.92; *p*=0.0010	F (1,8) = 8.82; *p*=0.017
Oas2		F (1,8) = 6.75; *p*=0.0316	
Gbp8	F (1,8) = 24.92; *p*=0.010	F (1,8) = 24.92; *p*=0.0010	F (1,8) = 24.92; *p*=0.0010
C4b	F (1,8) = 24.92; *p*=0.001	F (1,8) = 25.92; *p*<0.0001	F (1,8) = 8.9; *p*=0.0174
**HT22 Downregulated**	**Co-culture**	**Inflammation**	**Interaction**
Stc1	F (1,8) = 25.92; p<0.0001	F (1,8) = 25.92; p<0.0001	F (1,8) = 25.92; p<0.0001
Susd1	F (1,8) = 25.92; p<0.0001	F (1,8) = 24.92; p=0.0010	F (1,8) = 24.92; p=0.0010
Selenbp1	F (1,8) = 27.00; p<0.0001	F (1,8) = 25.92; p<0.0001	F (1,8) = 27.00; p<0.0001
Nlrc3	F (1,8) = 11.90; p=0.0086		
Gpr35	F (1,8) = 24.92; p=0.0010	F (1,8) = 24.92; p=0.0010	F (1,8) = 24.92; p=0.0010
Mlycd	F (1,8) = 24.92; p=0.0010	F (1,8) = 16.51; p=0.0036	F (1,8) = 24.92; p= 0.0010
Dok3			F (1,8) = 16.75; p=0.0034
Abcb1a	F (1,8) = 17.75; p=0.003		
St6gal1	F (1,8) = 17.00; p=0.0033		
Milr1	F (1,8) = 25.92; p<0.0001	F (1,8) = 25.92; p<0.0001	F (1,8) = 25.92; p<0.0001
Hpg	F (1,8) = 27.00; p<0.0001	F (1,8) = 27.00; p<0.0001	F (1,8) = 27.00; p<0.0001
Gpr56		F (1,8) = 16.75; p=0.0034	F (1,8) = 8.9; p = 0.017
Il23a		F (1,8) = 24.92; p=0.0010	F (1,8) = 24.92; p=0.010
Mettl7b			

Serpine1		F (1,8) = 24.92; p=0.0010	
BV2 Upregulated	**Co-culture**	**Inflammation**	**Interaction**
Ly6a	F (1,8) = 27.00; p<0.0001	F (1,8) = 25.92; p<0.0001	F (1,8) = 25.92; p<0.0001
Iigp1		F (1,8) = 24.92 p=0.0010	
Gbp8		F (1,8) = 24.92 p=0.010	
Cxcl9			
Serpine3G			
Fam26f	F (1,8) = 27.00; p<0.0001	F (1,8) = 25.92; p<0.0001	F (1,8) = 25.92; p<0.0001
Ly6c1	F (1,8) = 25.92 p<0.0001	F (1,8) = 24.92 p=0.0010	F (1,8) = 13.838 p= 0.0058
Gbp2	F (1,8) = 24.92 p=0.0010		
Cxcl11	F (1,8) = 27.00 p<0.0001	F (1,8) = 27.00 p=0.0010	F (1,8) = 17.78 p= 0.0029
Gbp4	F (1,8) = 8.52 p=0.019	F (1,8) = 25.90 p<0.0001	F (1,8) = 6.48 p= 0.034
H2-Aa	F (1,8) = 25.92 p<0.0001	F (1,8) = 27.00 p<0.0001	F (1,8) = 25.92 p=0.0009
Slamf8			
Gbp5		F (1,8) = 24.92 p<0.0001	
Zbp1		F (1,8) = 24.92 p=0.0010	F (1,8) = 24.92 p=0.0010
BV2 downregulated	**Co-culture**	**Inflammation**	**Interaction**
Cxcr2		F (1,8) = 25.92; p<0.0001	F (1,8) = 25.92; p<0.0001
Adm	F (1,8) = 27.00; p<0.0001	F (1,8) = 27.00; p <0.0001	F (1,8) = 27.00; p<0.0001
Lix1	F (1,8) = 17.51 p=0.0030		
Gpr183	F (1,8) = 24.92 p=0.0010	F (1,8) = 24.92 p=0.0010	F (1,8) = 24.92 p=0.0010
Myo7c	F (1,8) = 25.92 p<0.0001	F (1,8) = 24.92 p=0.010	F (1,8) = 25.92 p <0.0001
C5ar1		F (1,8) = 24.92; p=0.010	F (1,8) = 27.00; p=0.010
Spns2			
Car6	F (1,8) = 25.92; p<0.0001	F (1,8) = 27.00; p<0.0001	F (1,8) = 27.00; p<0.0001
Kif17			
Sla2	F (1,8) = 25.92 p<0.0001	F (1,8) = 25.92 p<0.0001	F (1,8) = 25.92 p<0.0009
Cxcl7	F (1,8) = 24.92; p=0.0010	F (1,8) = 24.92; p=0.0010	F (1,8) = 24.92; p= 0.0010
Chst1	F (1,8) = 27.00; p<0.0001	F (1,8) = 27.00; p<0.0001	F (1,8) = 25.92; p<0.0001
Padi2		F (1,8) = 25.92 p<0.0001	F (1,8) = 25.92 p<0.0009
Gpr97	F (1,8) = 27.00 p<0.0001	F (1,8) = 25.92 p<0.0001	F (1,8) = 27.00 p=0.0008

Table S3. Effect of inflammation and co-culture on gene expression.

HT22 Upregulated genes				
Culture type	***Standard***		***Co-culture***	
Inflammation	***no***	***yes***	***no***	***yes***
Cxcl9	1±0,15	6,94±0,99 [a]	0,84±0,28 [c]	**13,33±1,87** [d]
Iigp1	1±0,16	61,48±9,77	0,53±0,22	71,23±14,43
Gbp5	1±0,07	39,37±8,82	5,08±3,14	53±3,58
Gbp11	1±0,11	3,18±0,18	0,7±0,18	2,41±0,3
Ifi44	1±0,02	60,4±16,11 [a]	0,15±0,04	**64,11±4,95** [b,d]
Cxcl11	1±0,02	4,49±0,57	0,95±0,02 [c]	**12,45±0,37** [b,d]
Igtp	1±0,13	81,32±12,04 [a]	0,71±0,17	**79,93±4,76** [b,d]
Ifi47	1±0,05	11,92±0,29 [a]	1,78±0,08 [c]	**16,23±0,15** [d]
Usp18	1±0,1	26,74±5,85	0,28±0,03	43,26±5,42
Ifit3	1±0,11	1,7±0,05	0,79±0,19	2,06±0,25
Oas2	1±0,07	1,49±0,07	0,84±0,33	1,11±0,11
Gbp8	1±0,14	1,81±0,16 [a]	0,86±0,19 [c]	**1,82±0,14** [b,d]
C4b	1±0,1	4,38±0,14 [a]	0,82±0,19	**3,31±0,39** [d]
HT22 Downregulated genes				
Culture type	***Standard***		***Co-culture***	
Inflammation	***no***	***yes***	***no***	***yes***
Stc1	1±0,08	0,22±0,05 [a]	6,08±1,42 [c]	**0,62±0,06** [b,d]
Susd1	1±0,09	0,6±0,02 [a]	7,66±0,12 [c]	**0,47±0,07** [b,d]
Selenbp1	1±0,04	0,16±0 [a]	0,38±0,12 [c]	0,03±0,01 [b]
Nlrc3	1±0,15	1,22±0,02	0,82±0,18	0,81±0,13
Gpr35	1±0,03	0,82±0,05 [a]	1,12±0,05 [c]	**0,38±0,02** [b,d]
Mlycd	1±0,1	0,37±0,08 [a]	0,28±0,11 [c]	**0,46±0,12** [b,d]
Dok3	1±0,13	1,23±0,12 [a]	1,25±0,24 [c]	**0,74±0,07** [b,d]
Abcb1a	1±0,14	1,29±0,1	0,88±0,17	0,8±0,16
St6gal1	1±0,05	0,93±0,19	0,74±0,18	0,49±0,18
Milr1	1,01±0,21	0,94±0,12 [a]	4,84±0,89 [c]	**2,1±0,24** [b,d]
Hpg	1±0,1	0,87±0,03 [a]	5,13±0,83 [c]	1,53±0,34 [b]
Gpr56	1±0,04	0,2±0,12	0,55±0,31	0,42±0,2
Il23a	1±0,04	0,95±0,04 [a]	1,15±0,03 [c]	**0,68±0,03** [b,d]
Mettl7b	1,01±0,24	0,97±0,01	1,23±0,74	0,84±0,15
Serpine1	1±0,1	0,52±0,12	0,85±0,07	0,72±0,07
BV2 Upregulated genes				
culture type	***standard***		***co-culture***	
inflammation	***no***	***yes***	***no***	***yes***
Ly6a	1±0,17	2,01±0,25	0,32±0,12	3,99±0,56
Iigp1	1±0,11	2,97±0,75	0,51±0,07	2,42±0,56
Gbp8	1±0,08	1,98±0,85	0,46±0,07	1,31±0,18

Cxcl9	1±0,15	17,5±10,1	0,41±0,05	2,12±0,1
Serpine1	1±0,02	6,58±3,63	0,39±0,05	1,8±0,06
Fam26f	1±0,04	9,68±2,63 [a]	0,12±0 [c]	**1,93±0,27 [b,d]**
Ly6c1	1±0,09	7,75±1,11	0,16±0,05 [c]	**3,59±1,12 [b,d]**
Gbp2	1,01±0,17	0,72±0,3	2,47±0,54	2,55±0,89
Cxcl11	1,18±0,81	11,17±5,26	0,02±0,01 [c]	0,18±0,1 [b]
Gbp4	1±0,01	91,75±11,09	0,51±0,15	66,38±17,41
H2-Aa	1±0,05	5,71±1,75 [a]	0,8±0,15 [c]	**1,46±0,19 [b,d]**
Slamf8	1±0,06	11,18±2,11	0,72±0,07 [c]	**4,9±0,59 [b,d]**
Gbp5	1±0,13	58,71±16	0,28±0,09	62,55±23,16
Zbp1	1±0,11	1,05±0,27 [a]	0,44±0,07 [c]	**1,37±0,16 [b,d]**
BV2 Downregulated genes				
Culture type	***Standard***		***Co-culture***	
Inflammation	***no***	***yes***	***no***	***yes***
Cxcr2	1±0,02	0,61±0,09	0,85±0,09	0,76±0,11
Adm	1±0,06	0,39±0,1 [a]	24,6±10,6	**0,13±0,03 [b,d]**
Lix1	1±0,04	1,04±0,3	1,39±0,15	1,68±0,36
Gpr183	1±0,05	0,51±0 [a]	0,87±0,02 [c]	**0,07±0,01 [b,d]**
Myo7c	1±0,08	3,02±0,55 [a]	0,68±0,01 [c]	**0,29±0,08 [b,d]**
C5ar1	1±0,03	0,92±0,11 [a]	0,45±0,02 [c]	1,2±0,17 [b]
Spns2	1±0,1	0,24±0,07	0,89±0,02	0,2±0
Car6	1±0,15	0,35±0,11 [a]	13,25±2,82 [c]	**0,01±0,01[d]**
Kif17	1,01±0,22	1,29±0,3	0,58±0,13 [c]	**0,23±0,05 [b,d]**
Sla2	1±0,05	0,68±0,03 [a]	3,02±0,53 [c]	**0,26±0,04 [b,d]**
Cxcl7	1±0,05	0,42±0,1	0,51±0,06	0,19±0,05
Chst1	1±0,08	2,44±0,41 [a]	7,63±1,11 [c]	**1,29±0,12 [b,d]**
Padi2	1±0,14	0,16±0,08 [a]	4,12±1,52 [c]	**0,9±0,08 [b,d]**
Gpr97	1±0,02	0,65±0,18 [a]	26,38±9,17	**0,19±0,07 [b,d]**

Fold change ± SEM of inflammatory marker genes in HT22 and BV2 cells compared with non-inflamed cells cultured in standard monolayer. The genes that were significantly modulated by both co-culture and inflammation are reported in bold. a: $p < 0.05$ inflamed standard-culture vs non-inflamed standard-culture; b: $p < 0.05$ non-inflamed co-culture vs inflamed co-culture; c: $p < 0.05$ non-inflamed standard-culture vs non-inflamed co-culture; d: $p < 0.05$ inflamed co-culture vs inflamed standard-culture.

Table S4. *Primer sequences used to evaluate mRNA expression levels of mouse genes. Sequences were found in literature as reported in the references or designed using pick primer tool in PubMed Nucleotide.*

Abcb1a	CGCTATGGCCGAGAAGATGT	CTCTCACCAACCAGGGTGTC	
Adm	CATCCAGCAGCTACCCTACG	TTCGCTCTGATTGCTGGCTT	
Btc	GTGAGCGAGTGGACCTGTTT	TGCAGACGCCGATGACTAAA	
C4b	GAACAAATGTGCAAGAGCACC	CCTGTAGAGCAGAGCCTCTAA	[47]
C5ar1	GGGATGTTGCAGCCCTTATCA	CGCCAGATTCAGAAACCAGATG	[48]
Car6	GAACATCGAGAAGCCAGGAGA	GTGGACGTCCTTAGGCAACA	
Chst1	CAGGAGCTTCAAAACGATTCCA	CACACAGCAGTTACCTTCCCT	
Cxcl11	AGGAAGGTCACAGCCATAGC	CGATCTCTGCCATTTTGACG	[49]
Cxcl7	TGCGCTGCAGATGTACGAAT	TCAGTGTGGCTATCACTTCCAC	
Cxcl9	AATGCACGATGCTCCTGCA	AGGTCTTTGAGGGATTTGTAGTGG	[50]
Cxcr2	CTTCCAGTTCAACCAGCCCT	CTTAATCCTGCAGTAGTTCTACGA	
Dok3	GGCAGGAAGTGACCGAGTTT	CACCAGGAGGTAGGGTCCTT	
Fam26f	CAACTGCAATGCCACGCTA	TCCAACCGAACACCTGAGACT	[51]
Gapdh	TGCACCACCAACTGCTTAG	GGATGCAGGGATGATGTTC	
Gbp11	GAAAGCTGAGGAAATGAGAAGAG	GCCTTTTCAATCAGTAAAGAGG	[52]
Gbp2	CTGCACTATGTGACGGAGCTA	CGGAATCGTCTACCCCACTC	[52]
Gbp4	AGCTAACGAAGGAACAAAAG	GATGTTATGTCCCAGTTGATG	
Gbp5	CTGAACTCAGATTTTGTGCAGGA	CATCGACATAAGTCAGCACCAG	[52]
Gbp8	ACATCTGTCCATGAACCATGAAG	AAACCGTGATTCTGTCCTGCC	[52]
Gbp8	ACATCTGTCCATGAACCATGAAG	AAACCGTGATTCTGTCCTGCC	[52]
Gpr183	GCCACCTTCTGATATTGACTGT	GTTGCCAGTGGGGTAGTGAA	
Gpr35	ATCACAGGTAAACTCTCAGACACCAACT	CTTGAACGCTTCCTGGAACTCT	[53]
Gpr56	ACGTGGGCTGTGTCATCTC	GGACTTTGATGGTGTAGTCACG	
Gpr97	GGCCTCCTGTTCTTTCTGCT	GTGTCATACTCGTGGCCCTC	
H2-Aa	CAACTTGGGAGTCTTGACTA	CAGGAGGGAAGATGTTGTC	[54]
Hpg	GAATGTGAGGCAGTGTGTGGG	CATAGAGCCACCGATGATGC	
Ifi44	GACAGATACCAGTTCGATTC	TTTTCTTGATCTTTGCCACC	[50]
Ifi47	GTGAGAAACAGACCCGGTAT	ATGCCTCCTGCCTTACTGAT	[55]
Ifit3	ACTCTTTGGTCATGTGCCGT	AGGACTTCGCCTCCTCTGAA	[56]
Igtp	CCCATGGATTTAGTCACAAAG	CACCAGCAGTCATAGATTTAG	[50]
Il23a	TGTGCCCCGTATCCAGTGTG	AAAAGCCAGACCTTGGCGGA	
Kif17	CAGACAGGCAGTGGGAAGTC	TTCTCTGCACACTGAACGCT	
ligp1	AACTGGGCTAGAGAGATGGC	ACTACTGCATGTGGCAGGAT	[23]
Lix1	GCCTCCCTTTGTGAGCTATG	GCCTGGCCTCAGCTCTACT	[57]
Ly6c1	TGGTGTCAGGAGGGAGCTGCTA	GGCATTTACCAAGCAGGGGC	[58]
Mettl7b	CGCTTCATTGTGGCTTACGG	TAGCACCAGGGTACAGACCA	
Milr1	AGTCAGGCCCCTACAAATGC	AGGGCAGCTCTCATCTTTGG	
Mlycd	TTCCAACAACATCCAGGGCA	TGGGTCAGGCTGATGGAGTA	
Myo7b	GACCAACTGCCAGCCCTATT	ATGCACAGCTCTCGGTCAAA	

Nlrc3	GTCAGCTGCTACAAGTCCGGGAC	GAGCCTCAGAGTGCTTCGGTATCC	[59]
Oas2	GGCTATTCATCTGGCTGGTC	TTTCGTTGGGTGTGAGGTTT	[60]
Padi2	AGATGATCCTGCGCACCAAA	CAAAGAACGGGTTCTCCACG	
Selenbp1	TGAGCCTCTGCTCGTTCC	TGGACCACACTTTGTGCATT	
Serpine1	GCACAACCCGACAGAGACAA	ATGAAGGCGTCTCTTCCCAC	
Serpine1	GCACAACCCGACAGAGACAA	ATGAAGGCGTCTCTTCCCAC	
Sla2	TGGCAAACCTTTCCCTTTCC	GGTTTTCCCTCTGCTGGACA	
Slamf8	CCTGGCTGGTCTCTTTGGG	CGTCAGTGCAAGCATCCTTC	[61]
Spns2	CCATCCTGAGTTTAGGCAACGTG	CACCTTTCTATTGAAGCGGTCGC	[62]
St6gal1	TCTTCGAGAAGAATATGGTG	GACTTATGGAGAAGGATGAG	[63]
Stc1	TTGACACTCAGGGAAAAGCA	TGGAAAGTCGAACACCTCCG	
Susd1	CCTGCACCTCCAACAAGGAA	TCTTCTAAGAGATCATCAGGGGTCT	
Usp18	CAAGGAACAGTCTGAAATACAC	CACAGTAATGACCAAAGTCAG	[50]
Zbp1	GGGTCCCAGCTGATGTTTCT	TGAAGCAAGCTGACTTCCCT	[64]

Sequences were found in literature as reported in the references or designed using pick primer tool in PubMed Nucleotide.

References

47. Castillo, E.; Leon, J.; Mazzei, G.; Abolhassani, N.; Haruyama, N.; Saito, T.; Saido, T.; Hokama, M.; Iwaki, T.; Ohara, T.; et al. Comparative Profiling of Cortical Gene Expression in Alzheimer's Disease Patients and Mouse Models Demonstrates a Link between Amyloidosis and Neuroinflammation. *Sci. Rep.* **2017**, *7*, 17762. https://doi.org/10.1038/s41598-017-17999-3.
48. Hernandez, M.X.; Namiranian, P.; Nguyen, E.; Fonseca, M.I.; Tenner, A.J. C5a Increases the Injury to Primary Neurons Elicited by Fibrillar Amyloid Beta. *ASN Neuro* **2017**, *9*, 1759091416687871. https://doi.org/10.1177/1759091416687871.
49. Yang, C.H.; Wei, L.; Pfeffer, S.R.; Du, Z.; Murti, A.; Valentine, W.J.; Zheng, Y.; Pfeffer, L.M. Identification of CXCL11 as a STAT3-Dependent Gene Induced by IFN. *J. Immunol.* **2007**, *178*, 986–992. https://doi.org/10.4049/jimmunol.178.2.986.
50. Nair, S.R.; Abraham, R.; Sundaram, S.; Sreekumar, E. Interferon Regulated Gene (IRG) Expression-Signature in a Mouse Model of Chikungunya Virus Neurovirulence. *J. Neurovirol.* **2017**, *23*, 886–902. https://doi.org/10.1007/s13365-017-0583-3.
51. Kasamatsu, J.; Azuma, M.; Oshiumi, H.; Morioka, Y.; Okabe, M.; Ebihara, T.; Matsumoto, M.; Seya, T. INAM Plays a Critical Role in IFN-γ Production by NK Cells Interacting with Polyinosinic-Polycytidylic Acid-Stimulated Accessory Cells. *J. Immunol.* **2014**, *193*, 5199–5207. https://doi.org/10.4049/jimmunol.1400924.
52. Clough, B.; Finethy, R.; Khan, R.T.; Fisch, D.; Jordan, S.; Patel, H.; Coers, J.; Frickel, E.-M. C57BL/6 and 129 Inbred Mouse Strains Differ in Gbp2 and Gbp2b Expression in Response to Inflammatory Stimuli in vivo. *Wellcome Open Res.* **2019**, *4*, 124. https://doi.org/10.12688/wellcomeopenres.15329.1.
53. Farooq, S.M.; Hou, Y.; Li, H.; O'Meara, M.; Wang, Y.; Li, C.; Wang, J.-M. Disruption of GPR35 Exacerbates Dextran Sulfate Sodium-Induced Colitis in Mice. *Dig. Dis. Sci.* **2018**, *63*, 2910–2922. https://doi.org/10.1007/s10620-018-5216-z.
54. Israelsson, C.; Kylberg, A.; Björklund, U.; Ebendal, T. Anti-Inflammatory Treatment of Traumatic Brain Injury with Rabeximod Reduces Cerebral Antigen Presentation in Mice. *J. Neurosci. Res.* **2015**, *93*, 1519–1525. https://doi.org/10.1002/jnr.23607.
55. Kurihara, M.; Otsuka, K.; Matsubara, S.; Sh.hiraishi, A.; Satake, H.; Kimura, A.P. A Testis-Specific Long Non-Coding RNA, LncRNA-Tcam1, Regulates Immune-Related Genes in Mouse Male Germ Cells. *Front. Endocrinol.* **2017**, *8*, 299. https://doi.org/10.3389/fendo.2017.00299.
56. Intlekofer, K.A.; Clements, K.; Woods, H.; Adams, H.; Suvorov, A.; Petersen, S.L. Progesterone Receptor Membrane Component 1 Inhibits Tumor Necrosis Factor Alpha Induction of Gene Expression in Neural Cells. *PLoS ONE* **2019**, *14*, e0215389. https://doi.org/10.1371/journal.pone.0215389.
57. Wakeling, E.N.; Fyfe, J.C. Lix1 Knockout Mouse Does Not Exhibit Spinal Muscular Atrophy Phenotype. *J. Hered.* **2011**, *102* (Suppl. 1), S32–S39. https://doi.org/10.1093/jhered/esr031.

58. Stier, M.T.; Spindler, K.R. Polymorphisms in Ly6 Genes in Msq1 Encoding Susceptibility to Mouse Adenovirus Type 1. *Mamm. Genome* **2012**, *23*, 250–258. https://doi.org/10.1007/s00335-011-9368-9.
59. Karki, R.; Man, S.M.; Malireddi, R.K.S.; Kesavardhana, S.; Zhu, Q.; Burton, A.R.; Sharma, B.R.; Qi, X.; Pelletier, S.; Vogel, P.; et al. NLRC3 Is an Inhibitory Sensor of PI3K-MTOR Pathways in Cancer. *Nature* **2016**, *540*, 583–587. https://doi.org/10.1038/nature20597.
60. Bao, Y.; Gao, Y.; Shi, Y.; Cui, X. Dynamic Gene Expression Analysis in a H1N1 Influenza Virus Mouse Pneumonia Model. *Virus Genes* **2017**, *53*, 357–366. https://doi.org/10.1007/s11262-017-1438-y.
61. Wang, G.; van Driel, B.J.; Liao, G.; O'Keeffe, M.S.; Halibozek, P.J.; Flipse, J.; Yigit, B.; Azcutia, V.; Luscinskas, F.W.; Wang, N.; et al. Migration of Myeloid Cells during Inflammation Is Differentially Regulated by the Cell Surface Receptors Slamf1 and Slamf8. *PLoS ONE* **2015**, *10*, e0121968. https://doi.org/10.1371/journal.pone.0121968.
62. Bougault, C.; El Jamal, A.; Briolay, A.; Mebarek, S.; Boutet, M.-A.; Garraud, T.; Le Goff, B.; Blanchard, F.; Magne, D.; Brizuela, L. Involvement of Sphingosine Kinase/Sphingosine 1-Phosphate Metabolic Pathway in Spondyloarthritis. *Bone* **2017**, *103*, 150–158. https://doi.org/10.1016/j.bone.2017.07.002.
63. Shin, B.; Kress, R.L.; Kramer, P.A.; Darley-Usmar, V.M.; Bellis, S.L.; Harrington, L.E. Effector CD4 T Cells with Progenitor Potential Mediate Chronic Intestinal Inflammation. *J. Exp. Med.* **2018**, *215*, 1803–1812. https://doi.org/10.1084/jem.20172335.
64. Zhao, X.; Xie, L.; Wang, Z.; Wang, J.; Xu, H.; Han, X.; Bai, D.; Deng, P. ZBP1 (DAI/DLM-1) Promotes Osteogenic Differentiation While Inhibiting Adipogenic Differentiation in Mesenchymal Stem Cells through a Positive Feedback Loop of Wnt/β-Catenin Signaling. *Bone Res.* **2020**, *8*, 12. https://doi.org/10.1038/s41413-020-0085-4.
